# Melt flow control on lithological and geochemical heterogeneity of the oceanic upper mantle

**DOI:** 10.1093/nsr/nwag130

**Published:** 2026-03-10

**Authors:** Hui-Chao Rui, Luc S Doucet, C Johan Lissenberg, Dong-Yang Lian, Jie Li, Peng-Jie Cai, Sheng-Min Lai, Jian-Xi Zhu, Hong-Ping He, Jing-Sui Yang

**Affiliations:** Institute of Mantle and Metallogenesis, State Key Laboratory of Critical Earth Material Cycling and Mineral Deposits, School of Earth Sciences and Engineering, Nanjing University, Nanjing 210023, China; State Key Laboratory of Deep Earth Processes and Resources, Guangzhou Institute of Geochemistry, Chinese Academy of Sciences, Guangzhou 510640, China; Guangdong Provincial Key Laboratory of Mineral Physics and Materials, Guangzhou Institute of Geochemistry, Chinese Academy of Sciences, Guangzhou 510640, China; Earth Dynamics Research Group, School of Earth and Planetary Sciences, The Institute for Geoscience Research (Tiger), Curtin University, Perth, WA 6845, Australia; School of Earth and Environmental Sciences, Cardiff University, Park Place, Cardiff CF10 3AT, UK; Institute of Mantle and Metallogenesis, State Key Laboratory of Critical Earth Material Cycling and Mineral Deposits, School of Earth Sciences and Engineering, Nanjing University, Nanjing 210023, China; State Key Laboratory of Deep Earth Processes and Resources, Guangzhou Institute of Geochemistry, Chinese Academy of Sciences, Guangzhou 510640, China; Institute of Mantle and Metallogenesis, State Key Laboratory of Critical Earth Material Cycling and Mineral Deposits, School of Earth Sciences and Engineering, Nanjing University, Nanjing 210023, China; Bureau of Natural Resources and Planning of Juxian, Rizhao 276500, China; State Key Laboratory of Deep Earth Processes and Resources, Guangzhou Institute of Geochemistry, Chinese Academy of Sciences, Guangzhou 510640, China; Guangdong Provincial Key Laboratory of Mineral Physics and Materials, Guangzhou Institute of Geochemistry, Chinese Academy of Sciences, Guangzhou 510640, China; Center for Advanced Planetary Science, Guangzhou Institute of Geochemistry, Chinese Academy of Sciences, Guangzhou 510640, China; State Key Laboratory of Deep Earth Processes and Resources, Guangzhou Institute of Geochemistry, Chinese Academy of Sciences, Guangzhou 510640, China; Guangdong Provincial Key Laboratory of Mineral Physics and Materials, Guangzhou Institute of Geochemistry, Chinese Academy of Sciences, Guangzhou 510640, China; Center for Advanced Planetary Science, Guangzhou Institute of Geochemistry, Chinese Academy of Sciences, Guangzhou 510640, China; University of Chinese Academy of Sciences, Beijing 100049, China; Institute of Mantle and Metallogenesis, State Key Laboratory of Critical Earth Material Cycling and Mineral Deposits, School of Earth Sciences and Engineering, Nanjing University, Nanjing 210023, China

**Keywords:** mantle heterogeneity, lithological structure, melt depletion, ophiolite

## Abstract

The Earth’s upper mantle is heterogeneous in lithology and geochemistry, as demonstrated by variations in both abyssal peridotites and fossil oceanic mantle peridotites. The scarcity of spatial relationships between these peridotites, however, hinders further interpretation of the origin of mantle diversity as well as corresponding geodynamic processes. Here, we report the petrographic and chemical data of peridotites from the first fresh drill core (∼1300 m) across a Tibetan ophiolitic mantle sequence. This mantle column shows a primarily heterogeneous lithological structure consisting of repetitive ‘layered’ lherzolite, harzburgite, and dunite. Lherzolite and harzburgite have experienced 10%–15% and 15%–25% melt depletion, respectively. Such depletion cannot be generated by conventional partial melting models alone, but also requires melt-peridotite interaction in the asthenospheric mantle. Our work provides a high-resolution snapshot of the lithological structure and chemistry of the uppermost oceanic mantle and offers a melt flow model within the asthenosphere to explain the lithological variability of mantle rocks found in both mid-ocean ridges and supra-subduction zones.

## Teaser

Drilling into a Tibetan ophiolitic mantle column reveals a layered lithological structure, highlighting the key role of meltperidotite interaction in generating upper mantle heterogeneity

## INTRODUCTION

The upper mantle is a fundamental constituent of the Earth system, and it has co-evolved continuously with overlying crust throughout its history [1–3]. Adiabatic melting of fertile mantle peridotite during upwelling of the asthenosphere beneath a mid-ocean ridge generates a basaltic oceanic crust and refractory mantle residue. Conventionally, the composition of the upper mantle has been most extensively investigated through the geochemistry of mid-ocean ridge basalts (MORBs) [1], based on the assumption of a homogeneous mantle. Direct constraints on the nature of the upper mantle are provided by petrological and geochemical studies on mantle rocks from abyssal peridotites [2–4], ophiolites [5,6], as well as mantle xenoliths [7]. It has been well demonstrated that the upper mantle is heterogeneous in terms of lithology, as well as isotopic, major, and trace element compositions. For instance, refractory, ancient harzburgite and relatively fertile lherzolite commonly coexist in certain ophiolites. These heterogeneities reflect an array of processes, including variable melt depletion, refertilization, metasomatism, and recycling of oceanic crustal material into the mantle during the plate tectonic cycle [4,8]. The magnitude and spatial scale of lithological and compositional variations in the upper mantle are critical to gaining insights into lithospheric evolution, as well as mass and heat cycling within the Earth’s interior [9,10]. However, most mantle samples represent discrete fragments of the mantle, causing their primary structure and spatial relationships to be lost, which has impeded in-depth investigations into the distributions, origins, and timing of lithological and compositional heterogeneities in the upper mantle.

Drilling mantle sections and recovering potentially continuous sections of upper mantle

rocks provide a solution to this problem [11]. To date, the deepest reported drill hole (IODP Expedition 399 Site U1601C) into the present mantle is 1268 m at the Mid-Atlantic Ridge, recovering highly serpentinized harzburgites [10]. Recently, drilling programs conducted on ophiolites demonstrated high potential to reveal detailed structural and compositional characteristics of the oceanic lithospheric mantle [5,12]. Xu *et al*. [12] report an ∼1400 m drill borehole (Luobusa Ophiolite Scientific Drilling Program) from one of the Tibetan Yarlung-Zangbo ophiolites (YZO) (Fig. S1). Mantle peridotites in these drill cores generally experienced extensive serpentinization [5,10,12], which significantly obscures primary geochemical compositions and provides a challenge to the study of primary mantle processes.

**Figure 1.**
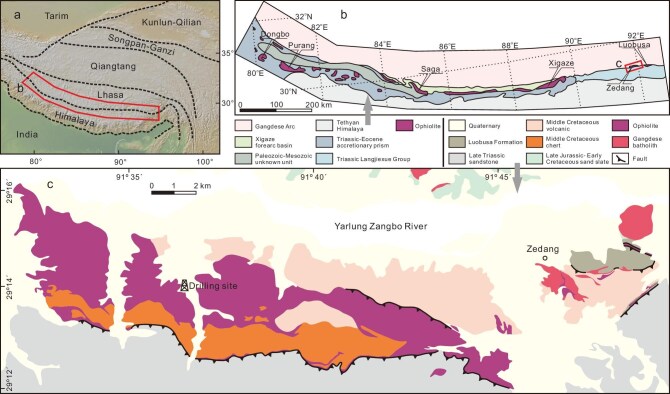
Simplified tectonic and geological maps showing the Zedang ophiolite in the Yarlung Zangbo Suture. (a) Major tectonic units of the Himalayan-Tibetan orogenic system, modified from Liu *et al*. [23]. (b) Simplified geological map of South Tibet showing the Yarlung-Zangbo Suture and major ophiolites, modified after Liu *et al*. [23]. (c) Geological map illustrating the Zedang ophiolite and adjacent tectonic units, modified after Xiong *et al*. [26].

The Zedang ophiolite, located in the eastern segment of Yarlung-Zangbo suture zone in Tibet (Fig. 1), is a massif representative of the YZO. The Zedang Ophiolite Scientific Drilling Program achieved a borehole (ZSD-1) depth of 1412.38 m with 96% core recovery [13]. Here, we report an integrated petrological and geochemical study of the Zedang ophiolite drill core, along with thermodynamic modeling of decompression melting and melt-peridotite interaction. We will use this high-resolution record to elucidate the geodynamic processes responsible for the heterogeneous structure and composition of the uppermost oceanic mantle.

### Drilling a fresh mantle section at the roof of the world

The Yarlung-Zangbo suture zone (YZSZ) extends east–west for more than 2000 km across southern Tibet, which separates the Tethyan Himalaya in the south from the Lhasa terrane in the north (Fig. 1a). The YZO discontinuously crop out along the YZSZ, and the main massifs from east to west are the Luobusa, Zedang, Xigaze, Saga, Dangqiong, Purang, and Dongbo ophiolites (Fig. 1b). These ophiolites represent the remnants of the Neo-Tethyan oceanic lithosphere [14–19]. One of the typical features of the YZO is the coexistence of lherzolite and (ultra-)refractory harzburgite [12,20–24]. The former generally has a mid-ocean ridge (MOR) geochemical affinity, whereas the latter spreads the fields of both MOR abyssal peridotite as well as forearc peridotites originated from a suprasubduction zone (SSZ). To this day, consequently, several competing geodynamic models have been proposed to explain these unique characteristics: (i) the geodynamic transition theory suggests lherzolites originate from anhydrous melting in the asthenospheric mantle beneath Neo-Tethyan (slow–ultraslow spreading) MOR, while harzburgites derive from hydrous melting in lithospheric/asthenospheric mantle during forearc extension at a SSZ [12,15,16,18,19]; (ii) the two-stage forearc accretion theory proposes YZO harzburgites form in mature subduction zones and lherzolites accrete later in the forearc during subduction initiation, resulting in a two-layered oceanic lithospheric mantle [22]; (iii) the YZO might represent an ocean core complex formed at slow–ultraslow spreading ridges and some refractory harzburgites probably represent ancient refractory mantle domains within the asthenosphere [25]; (iv) Additionally, Xiong *et al*. [24] observed a gradual transition from harzburgites to lherzolite across a 2 km geological profile in the Luobusa mantle, suggesting varying extents of melt-peridotite interaction and depletion with distance from the spreading center axis of MOR or SSZ.

The Zedang ophiolite (∼45 km^2^) crops out in the eastern segment of the YZSZ, sandwiched between the Triassic Langjiexue Group in the south and the Jurassic Gangdese arc complex in the north (Fig. 1b). This ophiolite has an incomplete litho-stratigraphy, consisting mainly of a mantle section (Fig. 1c) intruded by scarce Early Cretaceous dykes of gabbro and pyroxenite. These mafic dykes yield magmatic zircon U-Pb ages clustering at ∼132–128 Ma [26,27], consistent with widespread mafic magmatism at ∼135–120 Ma in the other YZO (see Liu *et al*. [28] and references therein). The mantle section comprises lherzolite and refractory harzburgite, with subordinary dunite lenses/dykes [22]. Although the origin of the YZO is still debated, the Zedang massif is likely to have originated from spreading centers in MOR, forearc, or back-arc basins of the Neo-Tethyan Ocean [25,29]. The Ophiolite Scientific Drilling Program in the Zedang targeted sites near the center of the massif (Fig. 1b). Unlike the drill core recovered from the Ophiolite Scientific Drilling Program in the Luobusa and the recent 1268 m deep hole along the Mid-Atlantic Ridge, where mantle peridotites are highly serpentinized [10,12], the drill core achieved in the Zedang provides a fresh mantle section preserved at the roof of the world, providing a unique opportunity to reconstruct upper mantle evolution.

## RESULTS

### Lithology and petrography

**Figure 2.**
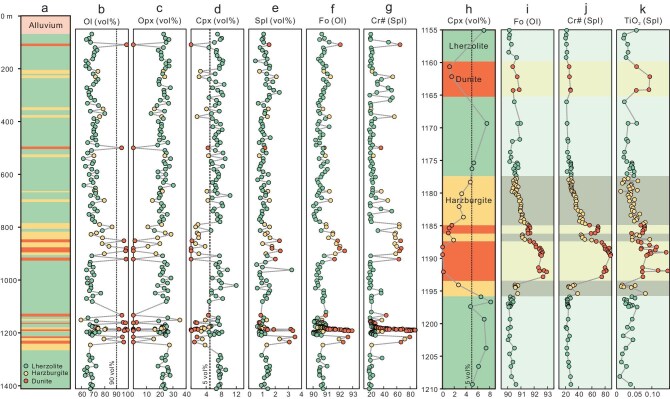
Lithological column of the Zedang ophiolite drill core and corresponding mineral modal and chemical variations. (a) Lithological column from 0 to 1412.38 m. (b–e) Modal variation of olivine, orthopyroxene, clinopyroxene, and spinel. (f) Olivine Fo values. (g) Spinel Cr# values. (h) Lithological column from 1155 to 1210 m, showing Cpx modal variation. (i) Olivine Fo values. (j) Spinel Cr# values. (k) Spinel TiO_2_ (wt%) concentration.

A total of 148 peridotite samples, systematically and evenly collected from the Zedang ophiolite drill core, were subjected to chemical analysis. Based on petrographic observations of over 1000 thin sections and calculated mineral modal compositions for 148 samples, a 1332.57 m lithological column (from 69.00 m to 1401.57 m depth, excluding alluvium) in the mantle sequence of the Zedang ophiolite drill core was established (Fig. 2a). Lherzolite, harzburgite, and dunite occur randomly within this mantle section, comprising 68.7%, 20.4%, and 10.9% of the total, respectively. Dunite is usually several meters to tens of meters thick. Complex lithological variations are evident in the depth intervals of 800–900 m and 1150–1250 m (Fig. 2). The transition from lherzolite to harzburgite is characterized by a gradual decrease in clinopyroxene (Cpx) modal compositions over scales of several meters. Conversely, contacts between dunite and adjacent lherzolite or harzburgite are marked by sharp modal variations of orthopyroxene (Opx) and Cpx over scales of ∼1 cm (Fig. 2; Fig. S2a).

Lherzolite exhibits porphyroclastic textures and comprises 62.1–79.9 vol% olivine (Ol), 13.5–30.3 vol% Opx, 5.0–12.0 vol% Cpx, and 0.5–3.3 vol% spinel (Spl) (Fig. S2a). Cpx porphyroclasts, up to 2–3 mm in diameter, host abundant exsolved Opx laths. Spl generally appears as anhedral grains, sometimes intergrown with fine-grained Cpx (Fig. S2b and c). Rare spinel-orthopyroxene-clinopyroxene symplectite (Fig. S2d) is observed only in lherzolite. Harzburgite contains 59.5–86.1 vol% Ol, 11.1–35.3 vol% Opx, 1.2–4.9 vol% Cpx, and 0.8–2.3 vol% Spl, displaying porphyroclastic textures (Fig. S2e). Opx porphyroclasts are commonly embayed by fine-grained Ol neoblasts. Cpx is mostly medium- or fine-grained (<1 mm) with few or no exsolution. Spl usually exhibits euhedral to subhedral morphologies (Fig. S2f–h). Dunite shows coarse-grained equigranular textures (Fig. S2h) and consists of 91.9–99.2 vol% Ol, 0–4.0 vol% Opx, 0–4.6 vol% Cpx, and 0.8–3.5 vol% Spl. Cpx appears as fine-grained (<0.5 mm) crystals and sometimes in contact with Spl or sulfide. Spl is rounded or euhedral and occasionally forms trails (Fig. S2i). Notably, the Zedang dunites are very different from IODP Expedition 399 Site U1601C dunites, which are typically tens of centimeters thick and lack clinopyroxene [10].

### Chemical composition

**Figure 3.**
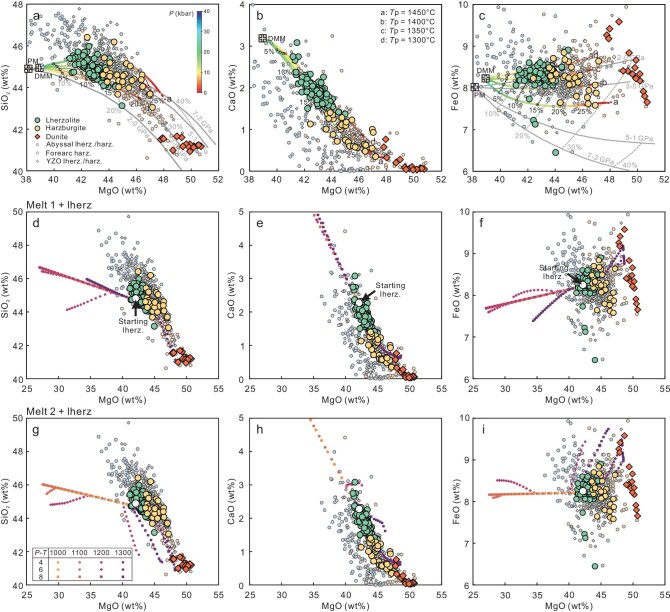
Whole-rock compositional variations of peridotites from the Zedang mantle column. Modeling results of decompressional melting of DMM (a–c) and melt-peridotite reaction (d–i) are shown. Grey lines (a, c) represent residues of polybaric fractional melting of primitive mantle at 2–0, 3–0, 5–1, and 7–2 GPa [52]. Data for YZO peridotites are compiled from Refs [24,44]. Data for abyssal peridotites are from Ref. [53] and forearc peridotites from Refs [8,54,55].

The majority of studied peridotites (89.8%) are fresh or weakly serpentinized, showing very low loss on ignition (LOI) values ranging from −0.18 wt% to 3.00 wt% (mean = 0.88 ± 0.72 wt%), with some outliers extending to 12.71 wt% (Fig. S3; Table S1). Although variations between LOI and whole-rock compositions suggest that serpentinization has little effect on most major oxides (Fig. S3), the whole-rock chemical compositions of samples with high LOI (>3%) are not considered in discussions of high-temperature mantle processes in this study. The whole-rock major element compositions of the peridotites are highly variable, encompassing the global range of abyssal and forearc peridotites (Fig. 3). The harzburgites have an intermediate content of total rare earth elements (ΣREE = 0.73–2.64 ppm, mean = 1.76 ± 0.67 ppm), which is lower than lherzolite (ΣREE = 2.46–8.57 ppm, mean = 5.08 ± 1.51 ppm) but higher than dunite (ΣREE = 0.21–5.68 ppm, mean = 1.34 ± 1.38 ppm). In addition, these peridotites commonly display concave-upward patterns on the chondrite-normalized REE diagram, showing variable enrichment of LREE compared with mantle residues after partial melting (Fig. S4; Table S2).

**Figure 4.**
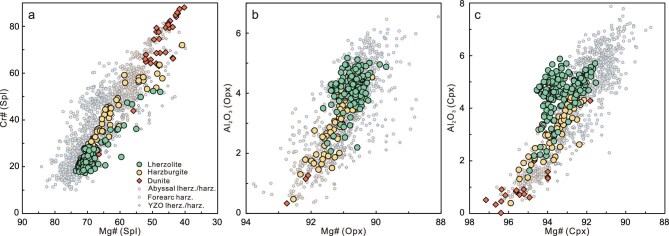
Mineral chemical compositional variations in the Zedang mantle column. (a) Spinel Cr# vs Mg#. (b) Orthopyroxene Mg# vs Al_2_O_3_ content. (c) Clinopyroxene Mg# vs Al_2_O_3_ content. Data for abyssal peridotites [4,43], forearc peridotites [8,54,56], and YZO peridotites [21–24] are shown for comparison.

Mineral chemical compositions exhibit a continuous variation from lherzolite to harzburgite and further to dunite (Fig. 2; Fig. 4; Tables S3–S5). For instance, the average Ol Fo [=100 × Mg/(Mg + Fe)] values show a progressive increase from lherzolite (90.5 ± 0.3) to harzburgite (90.9 ± 0.4) then to dunite (91.9 ± 0.7). Similarly, the average Spl Cr# [=100 × Cr/(Cr + Al)] and Mg# [= 100 × Mg/(Mg + Fe)] values in lherzolite, harzburgite, and dunite are 23.7 ± 7.0 and 69.0 ± 4.8, 43.8 ± 16.0 and 59.3 ± 9.0, and 61.1 ± 18.9 and 51.1 ± 8.3, respectively. The average Opx Al_2_O_3_ concentrations decrease from 4.1 ± 0.6 wt% in lherzolite to 2.6 ± 1.1 wt% in harzburgite, and to 1.8 ± 1.6 wt% in dunite, along with an increase of average Opx Mg# values from 90.8 ± 0.3 to 91.4 ± 0.5 and finally to 91.8 ± 1.0. Cpx shows a similar Mg#-Al_2_O_3_ trend (Fig. 4). Furthermore, Cpx in the lherzolite and harzburgite are low in ΣREE (1.95–5.70 ppm) and display spoon-shaped REE patterns with slight LREE enrichments (Fig. S5). In summary, Zedang lherzolite and harzburgite cover the field of both abyssal peridotite and forearc peridotite in whole-rock (Fig. 3; Fig. S4) and mineral chemistry (Fig. 4; Fig. S5).

**Figure 5.**
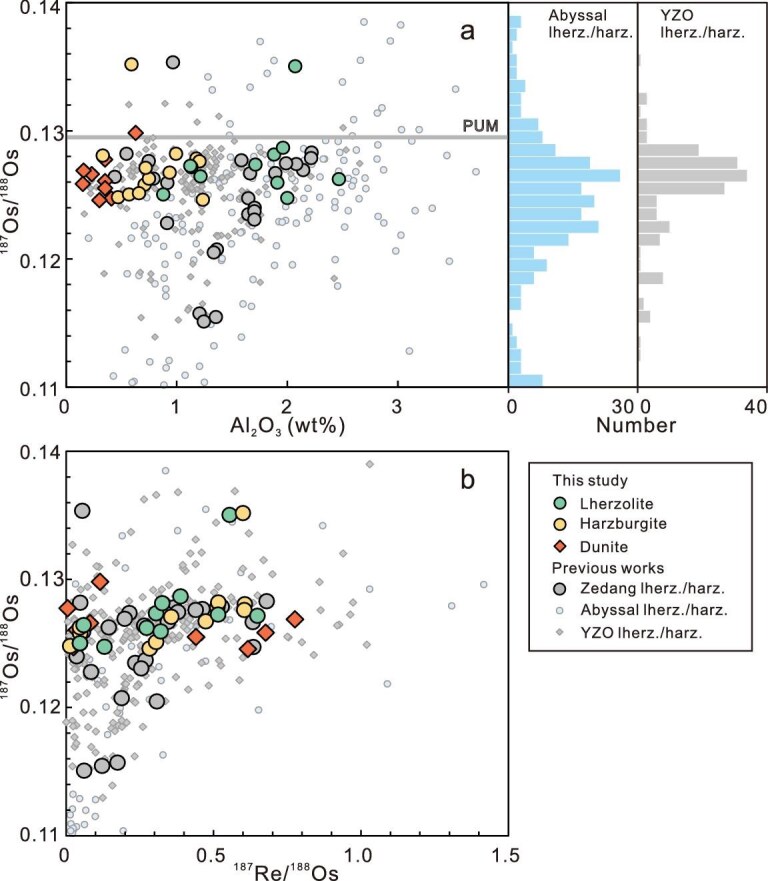
Re-Os isotopes of peridotites from the Zedang mantle column. (a) ^187^Os/^188^Os vs whole-rock Al_2_O_3_ content. (b) ^187^Os/^188^Os vs ^187^Re/^188^Os for the Zedang peridotite. Literature data of the Zedang peridotite are from Refs [29,40,44]. Abyssal peridotite data are compiled from Refs [9,42]. YZO data are from Liu *et al.* [49] and references therein.

The ^187^Os/^188^Os ratios of the analyzed Zedang samples, ranging from 0.12457 to 0.13516 (with the exception of a value of 0.14764 from a metasomatized dunite), overlaps with peak clusters observed in both abyssal and YZO peridotite in previous studies (Fig. 5a; Table S6). Notably, the Zedang peridotites exhibit highly variable whole-rock Al_2_O_3_ contents but relatively constant ^187^Os/^188^Os ratios (Fig. 5a). In addition, their ^187^Os/^188^Os ratios show no significant correlation with ^187^Re/^188^Os ratios (Fig. 5b) or Re and Os abundances (Fig. S6a and b).

## DISCUSSION

### Lithological structure of the uppermost oceanic mantle

To our knowledge, although the ∼1.3 km drill core of the Zedang ophiolite is not as deep as the core from the Luobusa ophiolite, the Zedang drill core represents the deepest drilling profile for any fresh peridotite massif worldwide (Fig. S1). It contains one of the freshest sections of mantle peridotite lithology and thus serves as an ideal relic of the uppermost oceanic mantle (Fig. 2). In contrast, the Oman ophiolite, is documented by several drilling profiles reaching ∼400 m depth and predominantly composed of serpentinized harzburgite [5], and the Horoman peridotite massif in Japan, is characterized by an ∼140 m layered sequence of plagioclase lherzolite to harzburgite [30].

**Figure 6.**
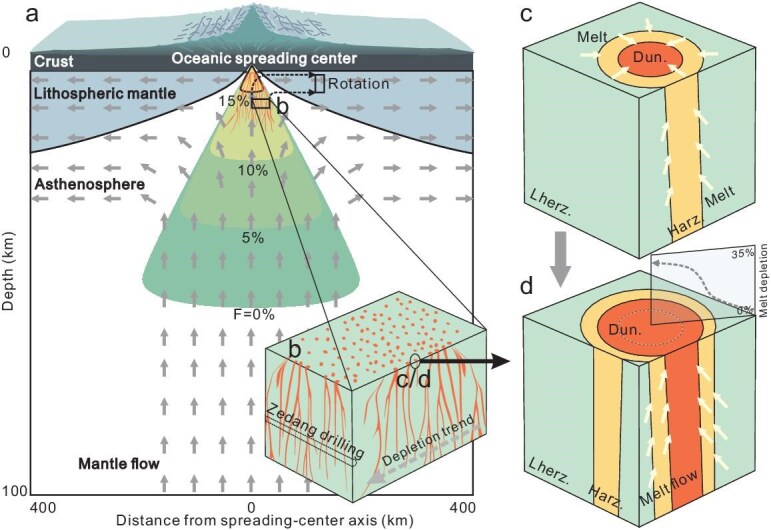
Cartoons illustrating the formation of the Zedang mantle column. (a) Basaltic melt is extracted from upwelling asthenospheric mantle flows beneath an oceanic spreading center. (b) A network of dunite melt channels and the reconstructed original orientation of the Zedang drill core. A depletion trend observed in the surface mantle section of the Luobusa ophiolite [24] is shown. During the interaction, primary lherzolites and harzburgites are transformed into harzburgites and dunites, respectively (c–d), thereby resulting in a gradual decrease in the extent of melt depletion with increasing radial distance from the dunite melt channels (d). The rotation of split asthenospheric columns with widespread melt channels leads to the ‘layered’ structure observed in the Zedang drill core.

Our study firstly uncovers the spatial and structural relationships of different lithologies in the Zedang mantle column, which presents a ‘layered’ occurrence of lherzolite, refractory harzburgite, and dunite (Fig. 2). The oceanic lithospheric mantle is theoretically produced through decompression melting and subsequent melt extraction from adiabatic upwelling of the asthenosphere (Fig. 6a), exhibiting a slightly upward depletion trend [2,31]. Tectonic processes (either faults or ductile flow) could be superimposed on this structure and juxtapose the different lithologies. However, the gradual mineralogical and compositional changes between the different lithologies (Fig. 2h–k), coupled with the absence of observed tectonic structures in the core, suggest that lithological variation primarily reflects intrinsic mantle structure rather than tectonic processes. Therefore, a 3D spatial network system of dunite surrounded by an aureole of depleted harzburgite within a lherzolite matrix can thus be reconstructed, based on the widespread occurrence of dunite lenses/dykes in surface outcrops [22] and ‘layered’ structure in the drill core of the Zedang ophiolite.

### Decompression melting cannot produce the repetitive ‘layered’ structure

Decompression melting and subsequent melt-rock interactions primarily govern the whole-rock and mineral chemical compositions of the lithospheric mantle [2,24,32]. We performed thermodynamic modeling of isentropic decompressional fractional melting of a depleted-MORB-mantle (DMM) source [33] (see the Methods for details and Table S7 for parameters). Thermodynamic modeling results (Table S8) suggest that variations in whole-rock major element compositions (Fig. 3) and heavy rare earth element (HREE) contents (Fig. S4d and e) in the majority of lherzolite and harzburgite can be reproduced after 10%–15% and 15%–25% partial melting, respectively, of the DMM source at depths ranging from 4 to 0 GPa. This is broadly consistent with the degrees of partial melting of 10% ± 2% and 15% ± 4% for lherzolite and harzburgite, respectively, calculated from Spl Cr# (F = 9 × ln(Cr#) + 23 [4]). It is important to note that the apparent degree of partial melting estimated from whole-rock geochemistry also considers melt depletion during partial melting and subsequent melt-peridotite interactions (details below).

However, such discordant degrees of melt depletion (10%–15% for lherzolite and 15%–25% for harzburgite) observed in the ∼1.3 km column of the Zedang massif cannot solely be explained by decompression melting. If we assume that the 1.3 km Zedang mantle column was originally vertical within the melting zone prior to its incorporation into the lithosphere, F variations in the lherzolites alone could yield a melt depletion gradient of ∼3.8%/km in this drill core. In comparison, our modeling results show the degree of partial melting across a 0.4 kbar (equivalent to 1.3 km in depth) exhibits only a negligible variation of 0.57%–0.29% under different potential temperature (Tp) conditions (Table S8), corresponding to a melt depletion gradient of 0.44%–0.22%/km. It means that mantle peridotite that has only experienced partial melting should display almost homogeneous lithology and major-element geochemistry within a limited mantle column. Besides, the Zedang drill core is likely to be approximately horizontal when restored to their original orientation within the melting zone (see below). In other words, the presence of layered lherzolites, harzburgite, and dunites in the mantle column of the Zedang ophiolite (Fig. 2) cannot be produced by partial melting alone.

### Widespread melt-peridotite interaction in the Zedang peridotites

Dunite occurrences in the mantle section are known to result from multi-stage melt-peridotite interactions, often serving as melt channels facilitating the transport of melt from the asthenospheric mantle to the oceanic crust beneath spreading ridges [31,34]. In this study, several lines of evidence indicate that dunites in the Zedang drill core have experienced intense interaction with migrating melt flows. First, spinels in these dunites exhibit a broader range of Cr# values (20.2–88.0) and distinctly higher TiO_2_ concentrations (0.03–0.31 wt%, mean = 0.10 ± 0.05 wt%) compared to the country lherzolites (mean = 0.04 ± 0.02 wt%) and harzburgites (mean = 0.06 ± 0.03 wt%). Furthermore, Ca concentrations in olivines from Zedang dunites range from 100 to 505 ppm (mean = 298 ± 132 ppm), which are significantly higher than those in lherzolites (mean = 131 ± 48 ppm) and harzburgites (mean = 136 ± 38 ppm) (Table S5). Given the incompatible behavior of Ti and Ca in peridotites during partial melting, the elevated Ti contents in spinels and Ca contents in olivines are aligned with interaction with migrating melts. Finally, fine-grained clinopyroxene is commonly present in Zedang dunites. Occasionally, Cpx crystals coexist with fine-grained spinels and sulfides, likely representing entrapped melt pockets. Collectively, we concluded that the Zedang dunite is closely related to interaction with migrating melts.

Several studies have noted that the formation of certain harzburgites is also likely associated with melt flows [18,24,32]. For example, based on investigations of chemical variations in reaction zones of the Trinity peridotite (Northern California, USA), Kelemen *et al*. [32] proposed that harzburgite can form through interaction between lherzolite and ascending basaltic melt in the lithospheric mantle. The fingerprints of melt-rock interaction and their linkage to the petrogenesis of lherzolite have long been overlooked. In this study, petrographic and geochemical features provide evidence that the melt-peridotite interaction is pervasive in some lherzolites and particularly harzburgites of the Zedang massif. For instance: (i) replacement of orthopyroxene by neoblasts of Ol + Cpx ± Spl in harzburgites (Fig. S2g); (ii) high spinel Cr# of certain harzburgites (up to 72.0) and lherzolites (up to 52.0); (iii) the melt depletion trend gradually increases with decreasing distance from the dunite channels (Fig. 2h–k); (iv) elevated Re concentrations (up to 0.61 ppb) and Re/Os ratios (up to 0.13) relative to the PUM (Re = 0.35 ppb, Re/Os = 0.09, Ref. [35]) in some harzburgites and lherzolites (Fig. S6c and d).

### Conversion of lherzolite to harzburgite during interaction with melt flow

We also performed thermodynamic modeling of melt-peridotite interaction under a series of P-T conditions (see Methods for details). Because the most fertile lherzolite samples from the Zedang drill core can be well reproduced by 10% partial melting of the DMM source, the residue of 10% decompression fractional melting at a Tp of 1350°C was chosen as the starting peridotite. Integrated melts generated by 15% (Melt 1) and 8% (Melt 2) fractional melting of the DMM source at Tp = 1350°C were chosen as the starting melts. Melt 1 represents typical MORB from mid-ocean spreading ridges, as well as forearc basalt (FAB) from forearc spreading ridges during the early stage of subduction initiation [36]. Melt 2 is derived from relatively low-degree partial melting of the DMM source and is more fertile than the melt equilibrated with the starting peridotite.

The modeling results show that temperature and pressure are the primary factors controlling the reaction pathways (i.e. depletion or refertilization) and composition of reactant products, whereas melt/rock ratios modulate the extent of reaction progression. The whole-rock major-oxide compositions of the Zedang lherzolite, harzburgite, and dunite can be accurately reproduced through the interaction of a lherzolite with mafic melts at a temperature of 1300°C and pressure of 4–6 kbar (Fig. 3d–i; Table S9), P-T conditions corresponding to the shallow asthenosphere beneath a spreading center. In these cases, clinopyroxene and orthopyroxene were consumed, and olivine precipitated during continuous interaction with mafic melts, leading to a gradual transformation of lherzolite to harzburgite and finally to dunite. Lower temperatures or higher pressures would trigger precipitation of plagioclase or/and clinopyroxene, resulting in depletion of MgO and enrichment of CaO in peridotites, which is not observed in our samples (Fig. 3).

Despite the low volume (10.9%) of dunite in this section, dunite occurs as lenses and dykes that construct a 3D spatial network within the asthenospheric mantle. Significantly, dunites are also identified in the asthenosphere by seismic anisotropy studies beneath spreading ridges [37]. These dunites are traditionally believed to serve as effective channels for focused melt flow [10,31]. The permeability of partially molten peridotite is closely related to the magnitude of melt flux and the velocity of melt extraction, and thus is a key property for understanding melt migration in the upper mantle. It has been suggested that melt flux is typically concentrated into high-permeability dunite channels in the asthenosphere [38]. The high permeability of dunite likely facilitates more melt percolation (high melt/rock ratio and high melt flux) and thus accommodates a more intense interaction. This intensification leads to conversion of harzburgite to dunite close to original dunite channels. Significantly, similar conversion of harzburgite to dunite by focused melt flow is observed in the Hole U1601C [10]. Meanwhile, the permeability of residual harzburgite proximal to newly formed dunite is expected to increase due to clinopyroxene and orthopyroxene dissolution [39]. Increased permeability of harzburgite thereby promotes interaction with neighboring lherzolite, leading to further depletion of the lherzolite and final conversion of lherzolite to harzburgite (Fig. 6b and c). These processes are consistent with observations that the extent of melt-peridotite interaction and melt depletion gradually decreases with increasing distance from the dunite, as revealed by the high-resolution chemical profile across the 1175–1190 m depth interval of the Zedang drill core (Fig. 2h–k). Therefore, we infer that permeability is important in explaining variable extents of melt depletion in the Zedang section.

It has been proposed that lherzolites in some ophiolites may form via transformation of refractory harzburgites through melt refertilization [40]. In this scenario, such lherzolites are expected to occur adjacent to dunite channels, which is inconsistent with the observations from the Zedang drill core. According to this permeability model, there should always be a dunite channel within harzburgite, as well as a harzburgite ‘layer’ between lherzolite and dunite. However, dunite is absent within certain harzburgite, and some dunite is in direct contact with lherzolite in the Zedang drill core (Fig. 2a). This discrepancy is likely due to insufficient sampling density. Alternatively, it implies that, in addition to partial melting and melt-peridotite interaction, other processes may also be involved in the formation of the Zedang mantle column. For example, numerous studies have indicated that previously depleted ancient mantle rafts, such as refractory harzburgites, are likely widespread in the asthenospheric mantle [9,41–43]. Nevertheless, the Zedang section provides unambiguous evidence that the lherzolite transforms to harzburgite and, ultimately, to dunite during interaction with melt flow within the asthenosphere.

### Re-Os chronometer records melt depletion in Neo-Tethyan mantle

Lherzolite, harzburgite, and dunite in the Zedang ophiolite display nearly identical ranges for subchondritic ^187^Os/^188^Os compositions with an average of 0.1265 ± 0.0014 (*n* = 31), which falls within the typical range of the depleted mantle as recorded by abyssal peridotites (Fig. 5a). Enrichment of Os (>10 ppb) in two dunite samples suggests the addition of S-saturated melt or fluid, consistent with findings reported for YZO peridotite by Xu *et al*. [44]. Additionally, two samples exhibit superchondritic ^187^Os/^188^Os ratios and were likely modified by recycled crustal materials [45,46]. The harzburgite influenced by low melt/rock ratio interactions exhibits an ^187^Os/^188^Os composition similar to the vast majority of dunite formed through high melt/rock ratio interactions, suggesting that melt-rock interaction in the asthenosphere and/or Os addition in the lithosphere do not appear to have significantly altered Os isotope compositions in the Zedang lherzolite and harzburgite. However, elevated Re concentrations and Re/Os ratios relative to the PUM in some Zedang peridotites deviate from the mantle depletion trends (Fig. S6c and d), indicating the addition of Re and subsequent radiogenic in-growth of ^187^Os in these samples. Importantly, the Zedang peridotite exhibits variable Al_2_O_3_ content but consistent ^187^Os/^188^Os ratios (Fig. 5a), a feature that can be explained by multi-stage melt depletion processes or a mantle source with heterogeneous Os isotopic compositions [44,45,47].

Rhenium-depletion model ages (T_RD_) of depleted peridotites can be used to estimate the minimum age of melt depletion events. Relative to the primitive upper mantle (^187^Os/^188^Os = 0.1296, Ref. [48]), the most depleted harzburgite and lherzolite samples (Re <0.2 ppb, Re/Os <0.02, Al_2_O_3_ <1.0 wt%) in the Re and Re/Os vs Al_2_O_3_ diagrams (Fig. S6c and d) yield T_RD_ ages ranging from 0.50 to 0.71 Ga, which is consistent with the reported T_RD_ peaks of depleted YZO harzburgite from previous studies [29,40,44]. It is noteworthy that Zedang and some YZO harzburgites record T_RD_ ages up to ∼2.0 Ga [40,44,45], indicating a heterogeneous mantle source with ancient Os isotope signatures derived from recycled sub-continental lithospheric mantle [21,44] or sub-oceanic mantle [45,49]. Despite rare samples sourced from ancient recycled mantle, it seems that the Zedang fertile lherzolite can be explained by decompression melting beneath the Neo-Tethyan MOR, and the depleted harzburgite by interaction with migrating melt flow during subduction, consistent with the geodynamic transition model from MOR to SSZ [12,15,16,18,19]. However, our modeling results suggest that the conversion of lherzolite to harzburgite and then to dunite occurs under P-T conditions corresponding to those of the shallow asthenospheric mantle, which is inconsistent with those of the lithospheric mantle modified during subduction in the geodynamic transition model. Alternatively, the Zedang peridotites, at least some of those recovered from the drill core, may originate from a protracted depletion event within the heterogeneous asthenospheric mantle beneath a spreading center. Subsequently, the Zedang oceanic lithospheric mantle likely underwent subduction-related modification and developed additional geochemical heterogeneity in an SSZ setting. For instance, the high Cr# spinel in specific dunites (Fig. 4a) may have resulted from modification by boninitic melts.

### New insight into the structure and evolution of the oceanic mantle

Mantle peridotites from Neo-Tethyan ophiolites exhibit substantial lithological and geochemical variations, which overlap the field of abyssal peridotites and straddle the region of forearc peridotites (Fig. 3; Fig. 4). Our study of the ∼1.3 km Zedang drill core first reveals spatial relationships and structures of these mantle peridotites. The observed lithological and geochemical variations record highly variable degrees of melt depletion processes, including decompression melting and melt-peridotite interaction within an upwelling asthenospheric column. Combined with modeling results and previous studies, we propose that the Zedang mantle section experienced a long-term evolutionary history in the asthenospheric mantle before it was incorporated into the oceanic lithosphere. The surface mantle section in the Luobusa ophiolite is thought to represent a vertical mantle column prior to its rotation and subsequent incorporation into the lithosphere [24]. The Zedang drill core is perpendicular to the vertical mantle column of the Luobusa massif and should therefore be approximately horizontal when reconstructed within the asthenospheric mantle beneath the spreading center, possessing an original orientation analogous to that of IODP Expedition 399 Site U1601C [10]. Within this asthenospheric mantle section, lherzolitic mantle experienced decompression melting and interacted with porous melt flow, leading to the formation of harzburgites. These harzburgites subsequently interacted with focused melt flow, resulting in the formation of dunites (Fig. 6a–c). When the asthenospheric column ascends, it bifurcates into two symmetrical parts that dynamically rotate within the mantle-flow regime, contributing to the formation of the juvenile uppermost oceanic mantle [24]. The rotation of these split asthenospheric columns, characterized by a widespread network of melt channels, helps explain the repetitive ‘layered’ structure observed in the Zedang column (Fig. 6d). In regard to previously proposed geodynamic models [12,15,16,18,19,22,24] considering lithological and geochemical variations reported in the YZO peridotite, therefore, our study uncovers important missing evolutionary processes within the asthenosphere of the Neo-Tethyan oceanic mantle.

As relics of the oceanic lithospheric mantle, Neo-Tethyan peridotites provide windows to understand mantle dynamics. Heterogeneity is a fundamental characteristic of the uppermost oceanic mantle [1,49], encompassing both lithological (e.g. lherzolite and refractory harzburgite) and isotopic geochemical variations [43,50]. Typical lithological heterogeneity is observed in dredged abyssal peridotites from mid-ocean ridges, consisting of dominant lherzolites and harzburgites with subordinary dunite [4,11]. This situation parallels the Neo-Tethyan oceanic mantle. Due to the scarcity of spatial relationships, the origin of these peridotites, especially harzburgites, is still enigmatic. For instance, some of the refractory harzburgites are believed to represent (i) recycled rafts of the ancient pre-depleted oceanic or continental lithospheric mantle [9,43], or (ii) residues of enhanced partial melting triggered by mantle thermal plumes or metasomatized, H_2_O-rich domains [51]. These models have great potential to explain the mantle heterogeneities observed in specific regions. In this study, the Zedang mantle column bridges the spatial gap between these lherzolites and depleted harzburgites, where dunite channels are surrounded by an aureole of depleted harzburgite within a lherzolite matrix (Fig. 6). In summary, our study provides a refined spatial structure for the variably depleted peridotites and depicts their detailed genetic links to melt flow, emphasizing that such harzburgite aureoles can be explained by melt-peridotite interaction within the asthenosphere. Furthermore, the distribution of different melt-depleted components in the present lithospheric mantle may mirror that observed in the Zedang column. Ultimately, like the Horoman and Oman peridotite massif [5,30], the Zedang column holds significant potential as a benchmark for understanding the oceanic mantle.

## METHODS

### Whole-rock major and trace elements

Whole-rock major oxides were measured using a Thermo ARL 9900 X-ray fluorescence (XRF) spectrometer at the State Key Laboratory for Mineral Deposits Research, Nanjing University. Before the major-element analysis, each 1.0 g of rock powder was fully mixed with 11.0 g of flux (49.75% Li_2_B_4_O_7_, 49.75% LiBO_2_, 0.5% LiBr). The mixed samples were melted at ∼1050°C and then quickly cooled as glass disks. A Chinese National ultramafic standard GBW07102, was repeatedly analyzed to monitor measurement procedure and data quality. Loss on ignition (LOI) was additionally measured on dried rock powder by heating in a preheated corundum crucible to 1050°C for 40 min and recording the percentage weight loss.

Whole-rock trace element compositions were obtained using a Thermo iCAP Qc inductively coupled mass spectrometry (ICP-MS) at the State Key Laboratory of Isotope Geochemistry, Guangzhou Institute of Geochemistry, CAS. The dissolution procedures of the samples were as follows: 40 mg rock powder, HNO_3_, HClO_4_, and HF were added to a Teflon bomb. The Teflon bomb was placed in a stainless-steel pressure jacket and heated at 190°C for >48 h, then opened and evaporated to dryness on a hotplate at 110°C. HNO_3_ was added, and the Teflon bomb was resealed and heated at 170°C for >4 h. The processed sample was diluted by a factor of 375 using 3% HNO_3_. A total of 0.75 g diluted sample, 1.60 g Rh-Re internal standard solution, and 5.65 g 3% HNO_3_ were mixed before measurements. The analytical accuracy was better than 10% for trace elements.

### Mineral major and trace elements

Mineral major element analyses were conducted using a JEOL JXA-8230 electron probe micro-analyzer (EPMA) at the State Key Laboratory for Mineral Deposits Research, Nanjing University. EPMA analyses were performed under an accelerating voltage of 15 kV and 20 nA beam current with a 2 μm beam spot. The counting times were 10 s and 5 s for peak and background elements, respectively. Natural mineral standards and a ZAF correction procedure were used for calibration. The relative standard deviations of the analyses on standards were within 1% for the major elements.

Mineral trace element compositions were analyzed using a Teledyne Cetac Technologies Analyte Excite laser ablation coupled with an Agilent Technologies 7900 quadrupole inductively coupled plasma mass spectrometer (LA-ICP-MS) at the CAS Key Laboratory of Mineralogy and Metallogeny, Guangzhou Institute of Geochemistry, CAS. For each analysis, laser ablation conditions of a 74–43 μm spot size, 6 Hz pulse frequency, and 4.5 J/cm^2^ energy fluence were applied. NIST 610 was used to correct the time-dependent drift of the sensitivity. BCR-2G and BIR-1G were used as the external calibration standards. Offline data processing was conducted using the ICPMSDataCal software with Si concentration (EPMA data) as an internal standard [57].

### Whole-rock Re-Os isotope

Whole-rock Re-Os isotope compositions were analyzed in the State Key Laboratory of Isotope Geochemistry, Guangzhou Institute of Geochemistry, CAS. About 0.5–2 g of each powdered sample was digested and equilibrated with ^185^Re- and ^190^Os-enriched spikes in reverse aqua regia (7.5 ml concentrated HNO_3_ + 2.5 ml concentrated HCl) for 48 h at 240°C in sealed Carius tubes [58]. Osmium was extracted by solvent extraction into CCl_4_ and back-extraction into concentrated HBr, with subsequent cleanup by microdistillation. The rhenium fraction was separated and purified using anion column chromatography.

Mass spectrometry procedures for the Os are given in Li *et al*. [59]. Os was loaded onto Pt filaments and measured as OsO_3_^−^ ions by negative-thermal ionization mass spectrometry (N-TIMS) using the electron multiplier mode on a Thermo-Finnigan Triton. Repeated analyses of the Os standard solution (DROsS) yield a mean ^187^Os/^188^Os value of 0.16094 ± 0.00007 (2σ, *n* = 12) for the period of analysis, and the values are in good agreement with the previously reported N-TIMS results [60].

Rhenium mass fraction was analyzed by inductively coupled plasma mass spectrometry (Thermo iCAP Qc). A conventional low-volume quartz impact bead spray chamber with a Peltier cooled (3°C) and a 0.4 ml/min borosilicate nebulizer (MicroMist GE) was used in the determinations. Ion lens settings, nebulizer gas flow rate, and torch position were optimized daily using a 10 ng/ml tuning In–Ce mixture standard solution in order to obtain high instrumental sensitivity and low oxide production levels. A peristaltic pump was not used, as free aspiration of the nebulizer provided better signal stability. The details of measurements are described elsewhere [59].

Total procedural blanks were 0.59 ± 0.16 pg (2σ, *n* = 4) with an ^187^Os/^188^Os ratio of 0.513 ± 0.082 (2σ, *n* = 4) on average for Os and 3.2 ± 0.6 pg (2σ, *n* = 4) for Re. All data were corrected for the procedural blank for each analytical batch. Blank contributions were generally insignificant. The average values of Re-Os isotope for basaltic reference material BIR-1 (^187^Os/^188^Os = 0.13385 ± 0.00035, Os = 0.319 ± 0.015 ppb, Re = 0.686 ± 0.004 ppb, 2σ, *n* = 4) are in good agreement with published data [61–63].

### Thermodynamic modeling

Isentropic decompressional fractional melting of the DMM source was modeled using the alphaMELTS 1.9 software package [64,65], at mantle potential temperatures (Tp) of 1300°C, 1350°C, 1400°C, and 1450°C. Open-system reactions of two types of mafic melts (Melt 1 and Melt 2) with the starting peridotite (relics after 10% melting of DMM source at Tp = 1350°C) have been modeled at the isobaric pressures of 4, 6, and 8 kbar and temperatures of 1000, 1100, 1200, and 1300°C. Melt 1 and Melt 2 represent integrated melts produced by 15% and 8% fractional melting (Tp = 1350°C) of the DMM source, respectively. Detailed parameters and results are shown in Tables S7–S9, and other modeling conditions are the same as those described by Xiong *et al*. [24].

## Supplementary Material

nwag130_Supplemental_Files
