# Supplementary material for: Melt flow control on lithological and geochemical heterogeneity of the oceanic upper mantle
Source: Natl Sci Rev. 2026 Mar 10;13(7):nwag130. doi: 10.1093/nsr/nwag130 (PMC13123518; doi:10.1093/nsr/nwag130)
Supplement: nwag130_Supplemental_Files [file nwag130_supplemental_files.zip › Supplementary Materials.pdf]

Supplementary Material for

Melt flow control on lithological and geochemical heterogeneity of the oceanic upper mantle

Hui-Chao Rui[1, 2, 3], Luc S. Doucet[4], C. Johan Lissenberg[5], Dong-Yang Lian[1*], Jie Li[2], Peng-Jie Cai[1], Sheng-Min Lai[1], Jian-Xi Zhu[2, 3, 6], Hong-Ping He[2, 3, 6, 7*], Jing-Sui Yang[1]

1 Institute of Mantle and Metallogenesis, State Key Laboratory of Critical Earth Material Cycling and Mineral Deposits, School of Earth Sciences and Engineering, Nanjing University, Nanjing 210023, China

2 State Key Laboratory of Deep Earth Processes and Resources, Guangzhou Institute of Geochemistry, Chinese Academy of Sciences, Guangzhou, 510640, China

3 Guangdong Provincial Key Laboratory of Mineral Physics and Materials, Guangzhou Institute of Geochemistry, Chinese Academy of Sciences, Guangzhou 510640, China

4 Earth Dynamics Research Group, School of Earth and Planetary Sciences, The Institute for Geoscience Research (Tiger), Curtin University, Perth, WA 6845, Australia

5 School of Earth and Environmental Sciences, Cardiff University, Park Place, Cardiff CF10 3AT, UK

6 Center for Advanced Planetary Science, Guangzhou Institute of Geochemistry, Chinese Academy of Sciences, Guangzhou 510640, China

7 University of Chinese Academy of Sciences, Beijing, 100049, China

*Corresponding authors: D-YL (ldy199008@163.com) & H-PH (hehp@gig.ac.cn).

Table of contents

Figure S1 to S6

Legends for Table S1 to S9

References

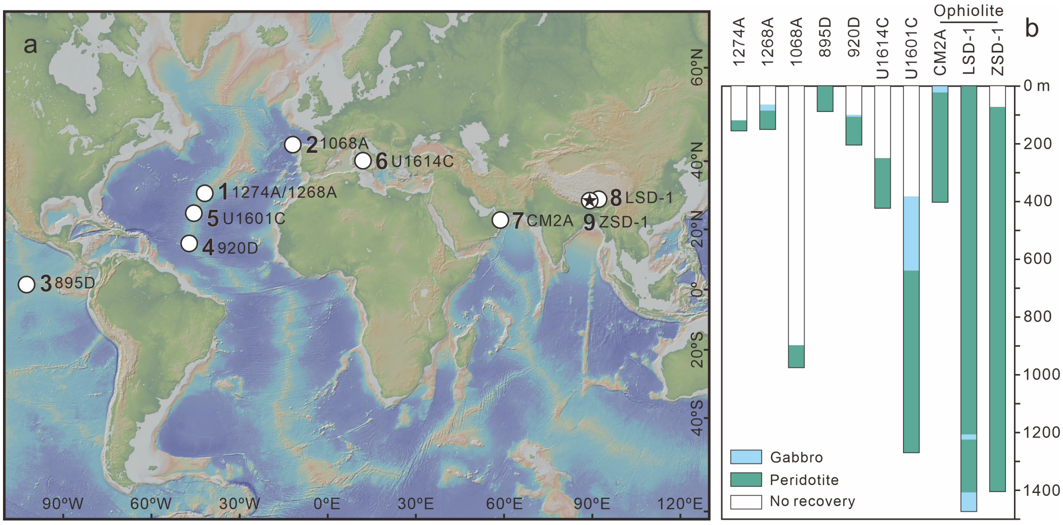

Figure S1. Locations of drilling sites recovering > 50 m thick oceanic mantle peridotite sections (a) and stratigraphy of the drill holes (b). 1-ODP Leg 209, 2-ODP Leg 173, 3-ODP Leg 147, 4-ODP Leg 153, and 5-IODP Expedition 399 are modified from ref.[1] and references therein. 6-IODP Expedition 402[2], 7-International Continental Scientific Drilling Program Oman Drilling Project[3], 8-Luobusa Ophiolite Scientific Drilling Program[4], 9-Zedang Ophiolite Scientific Drilling Program (this study).

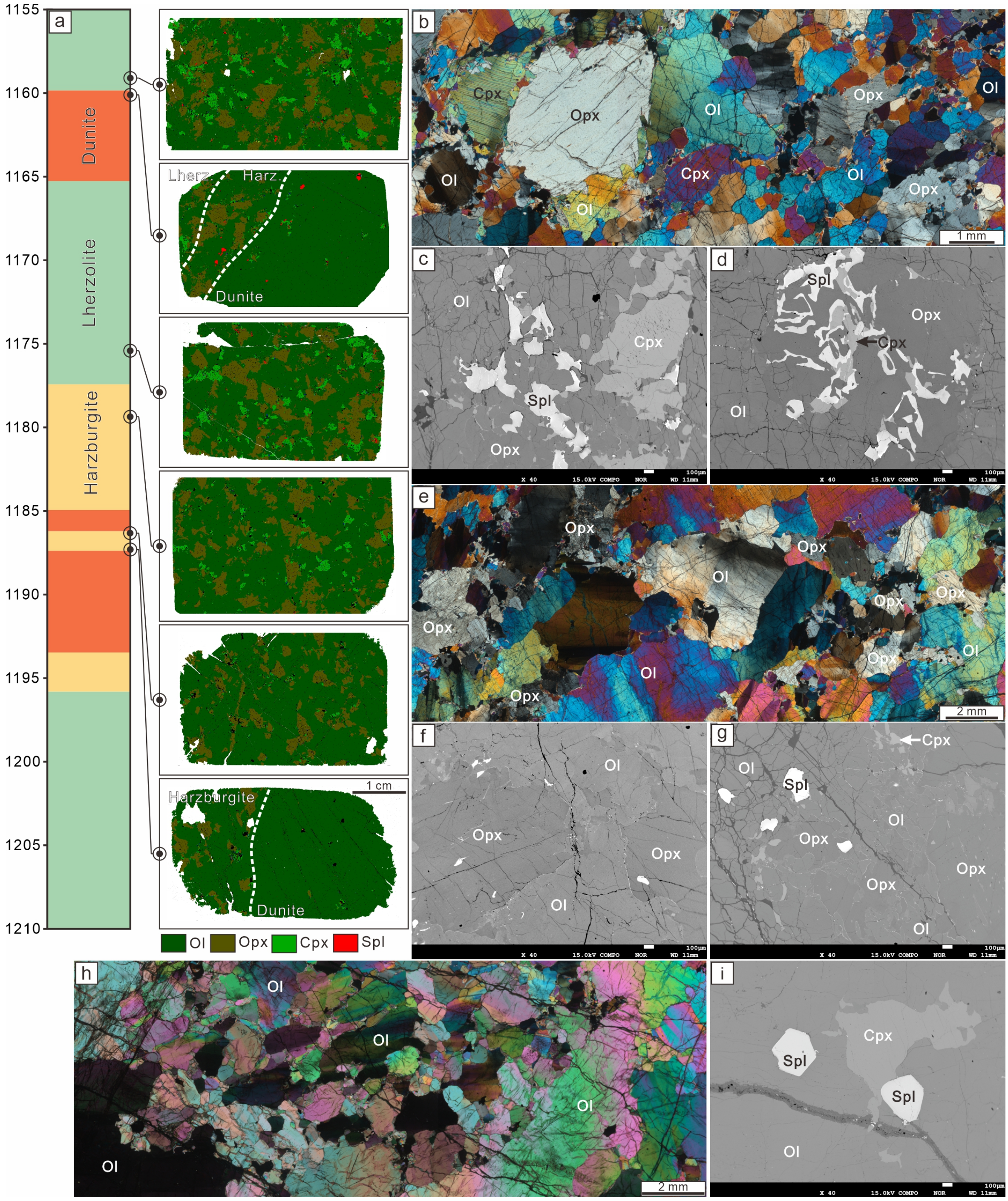

Figure S2. Petrographic characteristics of peridotites from the Zedang mantle column. (a) Representative Tescan Integrated Mineral Analyzer (TIMA) phase mapping. (b, e, h) Photomicrographs under crossed-polarized light and (c–d, f–g, i) backscattered electron images of lherzolite (b–d), harzburgite (e–g), and dunite (h–i). (b) Lherzolite exhibiting porphyroblastic textures. (c) Anhedral spinel grains. (d) Spinel-orthopyroxene-clinopyroxene symplectite. (e) Harzburgite displaying porphyroblastic textures. (f) Opx porphyroblasts commonly embayed by fine-grained Ol neoblasts. (g) Medium- or fine-grained clinopyroxenes with few or no exsolution. (h) Dunite showing coarse-grained equigranular textures. (i) Fine-grained crystals of anhedral clinopyroxenes and euhedral spinel.

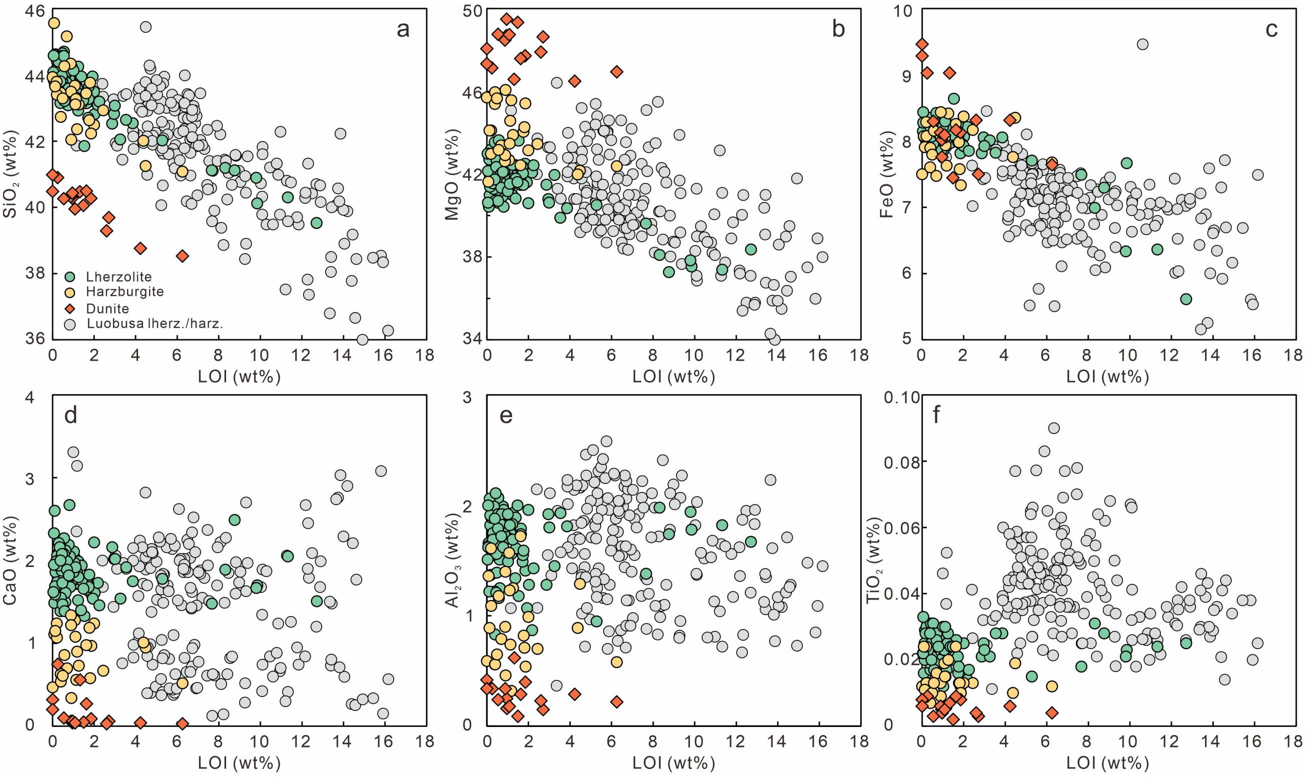

Figure S3. Co-variation plot of whole-rock LOI vs. major oxides of peridotites from the Zedang and Luobusa drill core. The Luobusa lherzolite and harzburgite data are from ref.[4].

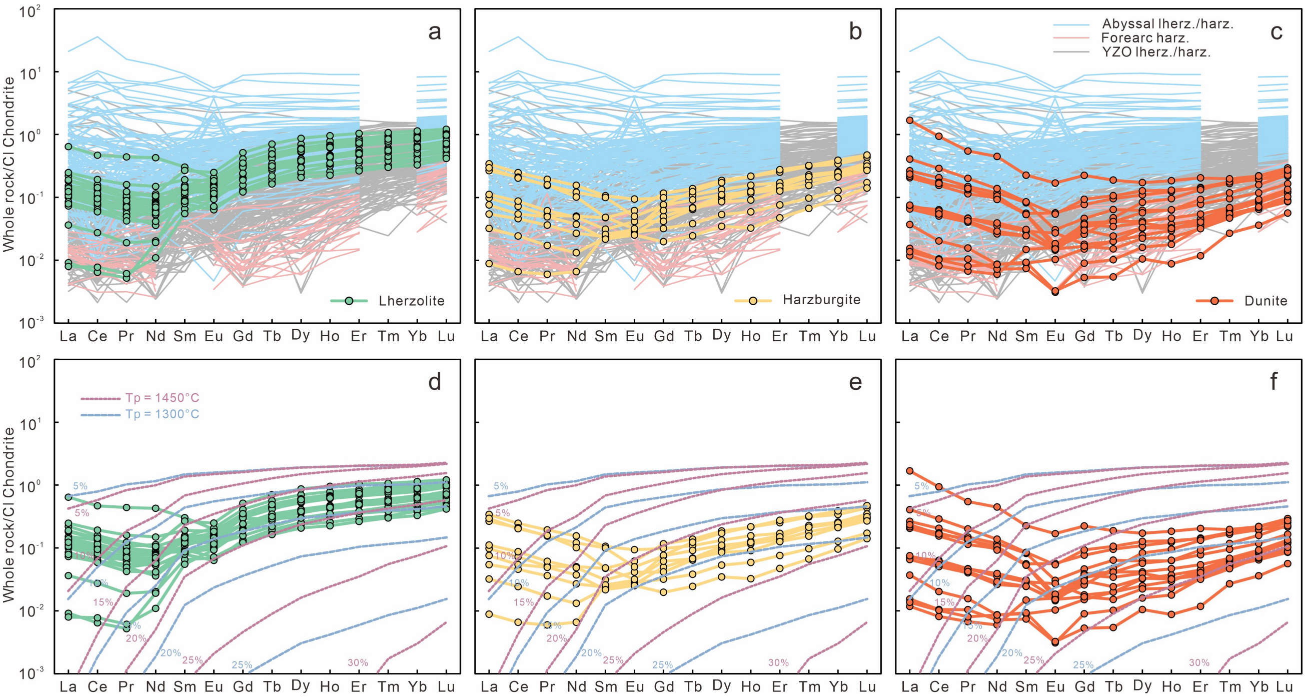

Figure S4. Chondrite-normalized REE patterns for the studied peridotite samples. Modeling results of decompressional melting of DMM are shown (d–f). Abyssal and forearc peridotite data are from ref.[5, 6], respectively. Normalizing values after ref.[7].

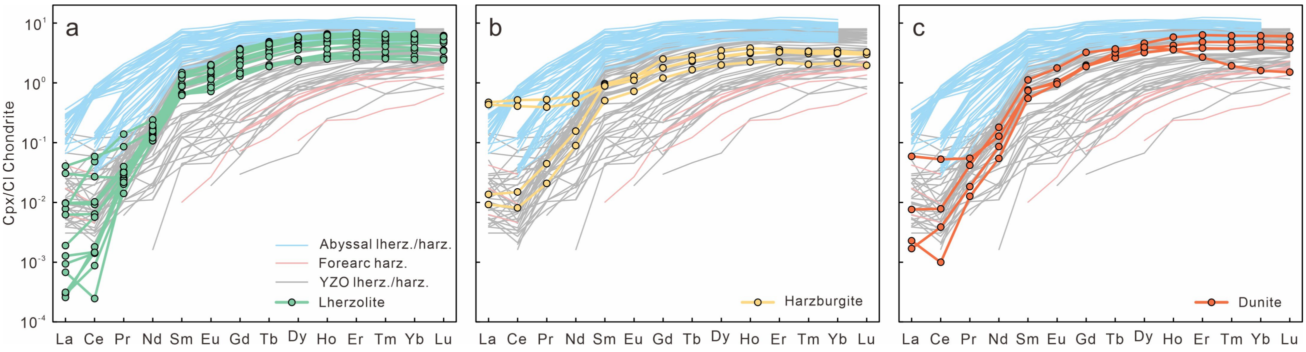

Figure S5. Chondrite-normalized REE patterns for the Cpx from studied peridotite samples. Abyssal and forearc peridotite data are from ref.[8, 9]. Normalizing values after ref.[7].

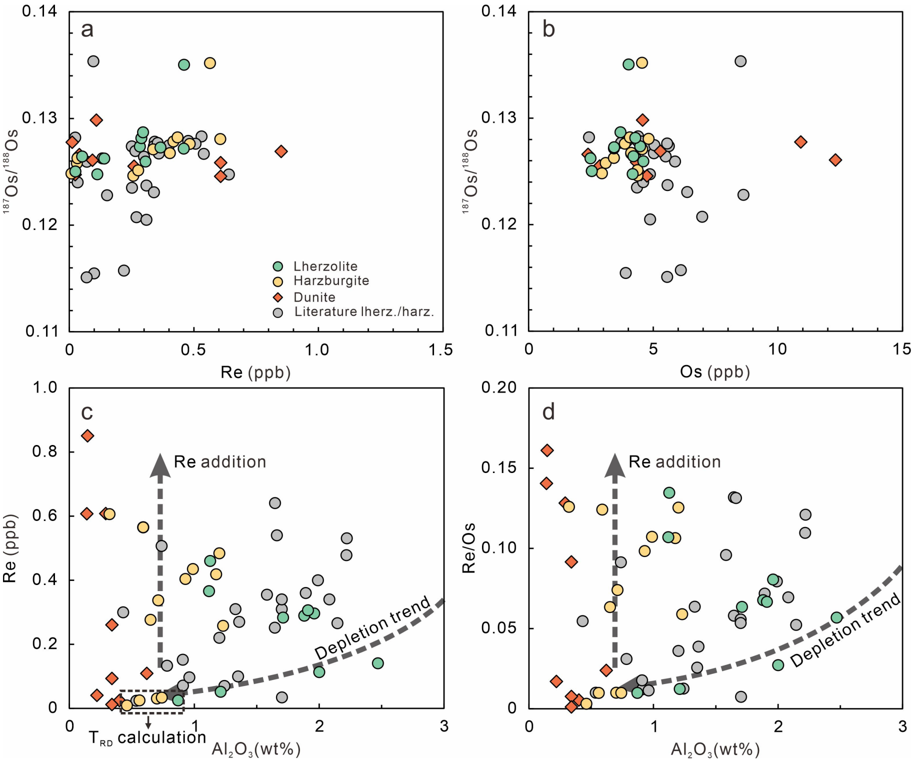

Figure S6. Re-Os concentration and Os isotopic compositional variation of peridotites from the Zedang ophiolite. Literature data of the Zedang lherzolite and harzburgite are from ref.[10-12].

Legends for Table S1 to S9

Table S1
Whole-rock major elements compositions (wt.%) and mineral modal (vol.%) of peridotite in the Zedang drilling core

Table S2
Whole-rock trace elements compositions (ppm) of peridotite in the Zedang drilling core

Table S3
Major elements compositions (wt.%) of olivine, orthopyroxene, clinopyroxene, and spinel of peridotite in the Zedang drilling core

Table S4
Trace elements compositions (ppm) of orthopyroxene and clinopyroxene of peridotite in the Zedang drilling core

Table S5
Trace elements compositions (ppm) of olivine of peridotite in the Zedang drilling core

Table S6
Whole-rock Re-Os isotopic compositions of peridotite in the Zedang drilling core

Table S7
Parameters used for alphaMELTS modeling of isentropic decompressional melting and melt-peridotite interaction

Table S8
Solid whole-rock results of alphaMELTS modeling of isentropic decompressional melting from the DMM source

Table S9
Solid whole-rock results of alphaMELTS modeling of melt-peridotite interaction

References

1. Lissenberg CJ, McCaig AM, Lang SQ *et al.* A long section of serpentinized depleted mantle peridotite. *Science* 2024; **385**: 623–629.
2. Sanfilippo A, Pandey A, Akizawa N *et al.* Heterogeneous Earth's mantle drilled at an embryonic ocean. *Nat Commun* 2025; **16**: 2016.
3. Kelemen PB, Leong JA, Carlos de Obeso J *et al.* Initial results from the Oman drilling project multi-borehole observatory: petrogenesis and ongoing alteration of mantle peridotite in the weathering horizon. *J Geophys Res-Solid Earth* 2021; **126**: e2021JB022729.
4. Xu X, Zoheir B, Yang J *et al.* Tectonized Neotethyan lithosphere in southeastern Tibet: results of the Luobusa ophiolite drilling. *Lithos* 2023; **436–437**: 106947.
5. Niu Y. Mantle melting and melt extraction processes beneath ocean ridges: evidence from abyssal peridotites. *J Petrol* 1997; **38**: 1047–1074.
6. Parkinson IJ, Pearce JA. Peridotites from the Izu–Bonin–Mariana forearc (ODP Leg 125): evidence for mantle melting and melt–mantle interaction in a supra-subduction zone setting. *J Petrol* 1998; **39**: 1577–1618.
7. McDonough WF, Sun SS. The composition of the Earth. *Chem Geol* 1995; **120**: 223–253.
8. Birner SK, Warren JM, Cottrell E *et al.* Forearc peridotites from Tonga record heterogeneous oxidation of the mantle following subduction initiation. *J Petrol* 2017; **58**: 1755–1780.
9. Lin K-Y, Warren JM, Davis FA. Trace elements in abyssal peridotite olivine record melting, thermal evolution, and melt refertilization in the oceanic upper mantle. *Contrib Mineral Petrol* 2023; **178**: 66.
10. Lai S, Yang J, Dilek Y *et al.* Petrological and Os isotopic characteristics of Zedong peridotites in the eastern Yarlung–Zangbo suture in Tibet. *Acta Geol Sin-Engl Ed* 2018; **92**: 442–461.
11. Xu Y, Liu J, Xiong Q *et al.* The complex life cycle of oceanic lithosphere: a study of Yarlung-Zangbo ophiolitic peridotites, Tibet. *Geochim Cosmochim Acta* 2020; **277**: 175–191.
12. Xu Y, Liu C-Z, Zhang C *et al.* Re-Os isotopic evidence for ancient melt depletion in refertilized Neo-Tethyan suboceanic mantle domain. *Chem Geol* 2025; **673**: 122520.
